# Regime Map of the Effective Medium Approximation Modelling of Micro-Rough Surfaces in Ellipsometry

**DOI:** 10.3390/s24041242

**Published:** 2024-02-15

**Authors:** Meijiao Huang, Liang Guo, Fengyi Jiang

**Affiliations:** Changchun Institute of Optics, Fine Mechanics and Physics, Chinese Academy of Sciences, Changchun 130033, China; huangmeijiao@ciomp.ac.cn (M.H.); guoliang@ciomp.ac.cn (L.G.)

**Keywords:** ellipsometry, rough surfaces, effective medium approximation, regime map

## Abstract

In this work, we discuss the precision of the effective medium approximation (EMA) model in the data analysis of spectroscopic ellipsometry (SE) for solid materials with micro-rough surfaces by drawing the regime map. The SE parameters ψ (amplitude ratio) and Δ (phase difference) of the EMA model were solved by rigorous coupled-wave analysis. The electromagnetic response of the actual surfaces with micro roughness was simulated by the finite-difference time-domain method, which was validated by the experimental results. The regime maps associated with the SE parameters and optical constants n (refractive index) and k (extinction coefficient) of the EMA model were drawn by a comparison of the actual values with the model values. We find that using EMA to model micro-rough surfaces with high absorption can result in a higher precision of the amplitude ratio and extinction coefficient. The precisions of ψ, Δ, n and k increase as the relative roughness σ/λ (σ: the root mean square roughness, λ: the incident wavelength) decreases. The precision of ψ has an influence on the precision of k and the precision of Δ affects the precision of n. Changing σ alone has little effect on the regime maps of the relative errors of SE parameters and optical constants. A superior advantage of drawing the regime map is that it enables the clear determination as to whether EMA is able to model the rough surfaces or not.

## 1. Introduction

Spectroscopic ellipsometry is a powerful technique for the determination of thin film structure and optical properties [1]. It is an essential tool in various fields of industry and research, in particular, materials and surface characterization techniques. Ma et al. [2] analyzed the optical parameters of graphene/MoS_2_ vdW heterostructure by spectroscopic ellipsometry in a broad band (1.3–5.0 eV). Fujita et al. [3] developed an ellipsometer equipped with a vacuum chamber and a vapor-controlling system to characterize the surface layers on the Si spheres. Leigh et al. [4] used ex situ SE spectra to develop an optical model for film characterisation, which was then applied to in situ data. They demonstrated that SE is a powerful tool for monitoring and optimisation of the critical early stages of polycrystalline diamond growth. Mandal et al. [5] characterized the sensor chip composed of partially embedded Au–Ag alloy nanoparticles using spectroscopic ellipsometry to determine the optical constant. 

Using spectroscopic ellipsometry to invert the optical constants of solid materials has the advantages of economy, speed, and being non-destructive [6]. The data analysis of spectroscopic ellipsometry is predicted by Fresnel’s equation under the assumption of a smooth surface [7]. In practice, the surface roughness of solid materials cannot be completely removed no matter how highly they are polished. Surface roughness will cause electromagnetic scattering of the incident light, which may affect the accuracy of the ellipsometric measurement [8,9,10,11]. 

The effective medium approximation (EMA) model, where the surface roughness layer is replaced by a single homogeneous and flat layer, is usually studied and applied by researchers to correct for the influence of rough surfaces in ellipsometry [12,13,14]. The root mean square (rms) roughness *σ* and the correlation length *τ* are used to characterize the morphologies of randomly rough surfaces. Volume fraction *f* and film thickness *d_EMA_* are the two main parameters of the EMA model used to characterize the rough layer of solid materials with rough surfaces. The rough layer of the surface is composed of materials and air and *f* is usually regarded as 50%. Therefore, to improve the precision of the EMA model, the relationship between *d_EMA_* and (*σ*, *τ*) of rough surfaces is usually studied by researchers. Koh et al. [15] researched amorphous semiconductor thin film materials with rough surfaces. They obtained the *σ* of the rough surface by atomic force microscopy and acquired the *d_EMA_* of the EMA model by real-time ellipsometry. They observed that *d_EMA_* is approximately equal to 1.5 times *σ*. Fujiwara et al. [16] found that the *d_EMA_* of the EMA model of a microcrystalline silicon thin film is approximately equal to 0.88 times *σ*. Petrik et al. [17] showed that the root mean square roughness of polycrystalline silicon, precipitated by chemical meteorology, also has a linear relationship with the film thickness. Yanguas-Gil et al. [18] calculated that *d_EMA_* is proportional to *σ*^2^/*τ*^α^. Liu et al. [19] reported that *d_EMA_* has a linear relationship with *σ*^2^/*τ* and the proportionality coefficient and intercept change with the materials. Yu et al. [20] found that the EMA model constructed with *d_EMA_* = *σ* + 0.80 *h* (*σ* represents the root mean square height and *h* represents the average height, respectively) better fits the SE parameters. Above all, using EMA to model rough surfaces has the drawback that the formulas of the film thickness are undefined and the precision of the model is difficult to determine.

In this work, in order to estimate the precision of the EMA model of the randomly micro-rough surfaces with different morphological parameters intuitively, we draw the regime maps about the relative errors of the SE parameters and optical constants. Specifically, the rigorous coupled-wave analysis (RCWA) method is utilized to solve the SE parameters of the EMA model. The finite-difference time-domain (FDTD) method, which is validated with experimental results, is applied to simulate the electromagnetic response from micro-rough surfaces. And the FDTD simulations are regarded as the “measured” data. Then we compare the SE parameters of the EMA model with the measured values and draw the regime maps associated with the relative errors of the SE parameters of the EMA model. The actual optical constants n and k are assumed to be 3.2 and 2.6, respectively. The results calculated from the EMA model are compared with the actual optical constants to plot the regime maps associated with the relative errors of utilizing the EMA model to deduce the optical constants of the randomly micro-rough surfaces.

The remainder of this paper is structured as follows: the models, simulation methods and experimental verification are presented in Section 2; the results and discussion are provided in Section 3; Section 4 concludes this paper.

## 2. Theoretical Background

### 2.1. Micro Randomly Rough Surface Model and EMA Model

Randomly rough surfaces are usually characterized by the root mean square height σ which represents the vertical scale and the correlation length τ which denotes the lateral scale. The autocovariance function of Gaussian distributed randomly rough surfaces is as follows [21]:

(1) $$\langle H\left(\vec{p}\right)H\left(\vec{p}+\vec{q}\right)\rangle =\sigma^{2}\exp\left[-\left(q^{2}/\tau^{2}\right)\right]$$

where $H(\vec{p})$ represents the height at position $\vec{p}$, $\vec{q}$ is a spatial vector, and *q* denotes its magnitude.

The EMA model can be used to simplify randomly rough surfaces whose morphologies are complex in ellipsometry. In detail, the surface roughness layer is replaced by a single homogeneous and flat layer, where the thickness of the layer is equal to *σ*. The Bruggeman formula takes the form [22]:

(2) $$f\frac{\varepsilon_{n}-\varepsilon_{eff}}{\varepsilon_{n}+2\varepsilon_{eff}}+(1-f)\frac{\varepsilon_{void}-\varepsilon_{eff}}{\varepsilon_{void}+2\varepsilon_{eff}}=0$$

where *f* represents the volume fraction of the measured sample and is regarded as being constant at 50%, $\varepsilon_{n}$ and $\varepsilon_{void}$ represent the dielectric function of the measured sample and void spaces, respectively, and $\varepsilon_{eff}$ is the effective dielectric function of the rough layer.

### 2.2. Simulation Methods of SE Parameters of EMA Model and Randomly Rough Surfaces

Based on the detailed surface geometrical morphology, the SE parameters of the EMA model and randomly rough surfaces can be obtained by solving Maxwell’s equations. In this work, the RCWA method and the FDTD method are employed to simulate the electromagnetic response of the EMA model and rough surfaces, respectively. 

The RCWA method, which has the advantages of high efficiency and low-memory requirement, has been widely applied to study the radiative characteristics of one-dimensional periodical structures [23,24,25,26]. The fundamental theory of the RCWA method is as follows [7]: the calculation region is divided into three parts including the grating region, incident region, and transmission region. The expressions of the incident region and transmission region can be solved according to Maxwell’s equations. The electromagnetic field and dielectric function of the grating region are expanded as Fourier series, while the electromagnetic fields of the incident region and the transmission region are expressed as Rayleigh expansions. Substituting the expanded parameters into the Maxwell or Helmholtz equations in combination with the boundary-matching conditions, the coupled-wave equations can be established and solved. Consequently, the diffraction coefficients and the electromagnetic fields of each order can be obtained and, then, the SE parameters of the EMA model will be determined. The detailed algorithms of RCWA have been published previously [23,24].

FDTD is a robust numerical method which requires a lower computing resource for the simulation of three-dimensional geometrical structures compared with the RCWA method. FDTD can directly solve the differential Maxwell equations in the time domain to accurately calculate the electromagnetic response [27]. The FDTD method scales with high efficiency on a parallel-processing CPU-based computer and performs especially well for three-dimensional geometrical structures. Therefore, the FDTD method is employed to simulate the SE parameters of randomly rough surfaces in this work. The more detailed algorithm of FDTD have been published previously [28,29].

### 2.3. Experimental Verification of the FDTD Method

**Figure 1:**
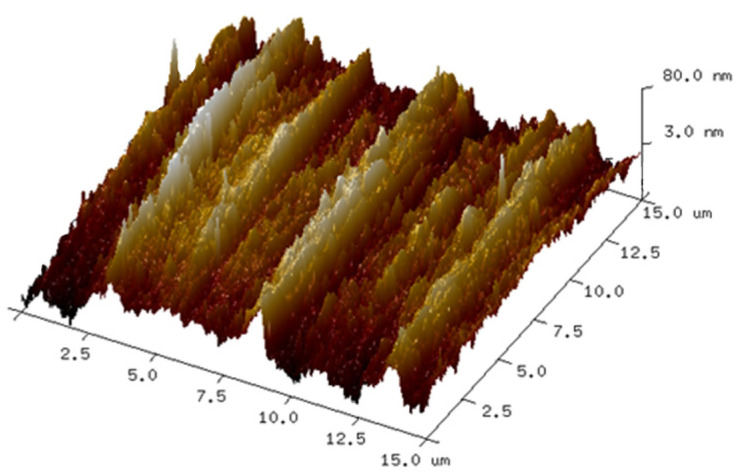
Morphology of the nickel alloy rough surface scanned by AFM.

The experimental verification of the FDTD method was performed on a nickel alloy material with a rough surface. The size of the sample was 22 mm × 18 mm × 1 mm. Atomic force microscopy (AFM), which can obtain information on surface morphology and roughness, is usually used to be an auxiliary surface characterization technique in the SE optical technique [30,31]. In this work, the surface geometrical morphology of the sample was scanned by AFM as shown in Figure 1. The scanning area of the sample was 15 μm × 15 μm and the resolution of AFM was 512 × 512. Then, the morphological parameters of the sample scanned by the AFM were input into FDTD to simulate the electromagnetic response and obtain the SE parameters, regarded as the numerical results. 

The experimental results of the sample were measured by an infrared variable-angle spectroscopic ellipsometer (IR-VASE, manufactured by J. A. Woollam Co., Inc., Lincoin, NE, USA). Specifically, a beam of linearly polarized light is emitted onto the surface of the sample at an oblique angle which was set to 60°. The incident wavelength λ was in the range of 1 μm ≤ λ ≤ 2 μm and the aperture size of the detector in the ellipsometer was 5 mm. The linearly polarized light is reflected by the surface of the sample and becomes elliptically polarized light. The light parallel to the incident plane is called “p-polarized light” and the light perpendicular to the incident plane is called “s-polarized light”. The parameter Ψ represents the angle whose tangent gives the ratio of amplitude variation upon reflection for p- and s-polarizations. The parameter Δ represents the difference in phase experienced upon reflection by p- and s-polarizations. The amplitude ratio Ψ and the phase difference Δ, which are known as the SE parameters, are defined by [20]:

(3) $$\rho =\tan\psi \;exp\left(i\Delta \right)=(\frac{E_{rp}}{E_{ip}})/(\frac{E_{rs}}{E_{is}})$$

where ρ is the ellipsometric ratio; E*_rp_* and E*_ip_* represent the reflected and the incident electric field intensity of p-polarizations, respectively; and E*_rs_* and E*_is_* denote the reflected and the incident electric field intensity of s-polarizations, respectively.

**Figure 2:**
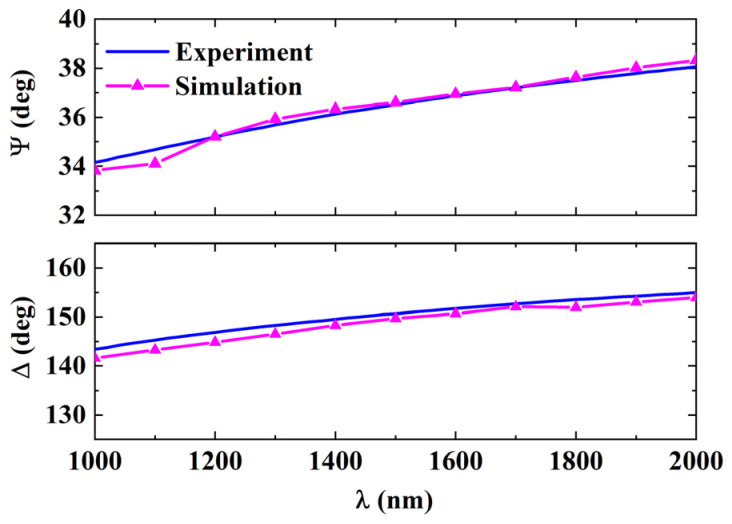
Comparison of the results between the simulation and the experiment.

A comparison of the simulated and measured SE parameters Ψ and Δ of the nickel alloy surface is shown in Figure 2, where the magenta dotted lines and solid blue lines represent the results of the simulation and experiment, respectively. When the wavelength was 1100 nm, the errors of Ψ and Δ obtained by numerical calculation were 1.47% and 1.37%, respectively, which were the maximum errors within the range of wavelengths. Therefore, the simulated results agreed well with the experimental results.

## 3. Results and Discussion

### 3.1. Selection of the Optical Constants of Actual Rough Surfaces

**Figure 3:**
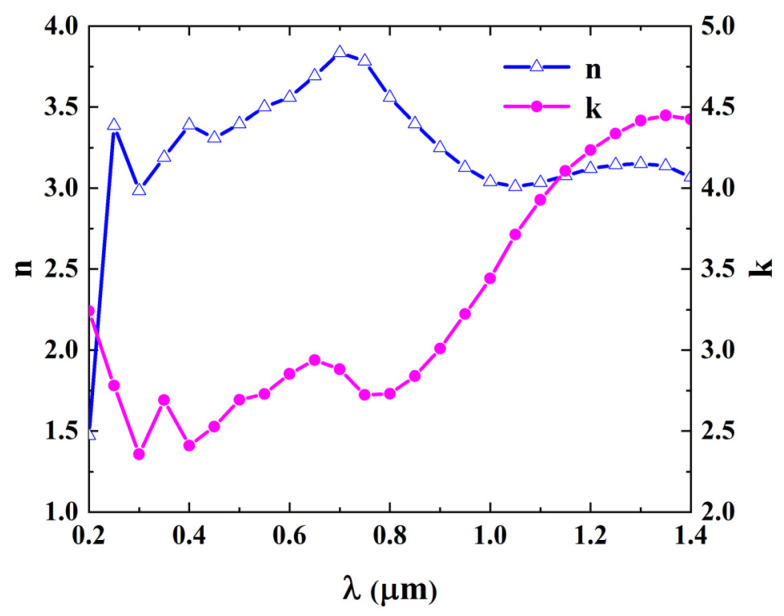
Optical constants of tungsten within the wavelength range of 0.2 μm ≤ λ ≤ 1.4 μm [33].

Tungsten (W) is a material with high absorption that has been widely used to design nano-structures [32]. The optical constants of W within the wavelength range of 0.2 μm ≤ λ ≤ 1.4 μm are shown in Figure 3 [33]. The optical constants n and k changed with the wavelength λ and the values of n and k were 3.2 and 2.6, respectively, at the wavelength of 0.35 μm. Actually, most of the optical constants of materials vary with λ. Ignoring the influence of wavelength and considering the computational efficiency, we chose the material whose optical constants n and k were 3.2 and 2.6, respectively, and remained unchanged to represent most materials with high absorption and researched the regime map of the EMA model of this material with rough surfaces in this work.

### 3.2. Coordinates of the Regime Map of the EMA Model

No matter how smooth it is polished, the surface of the materials has roughness. Generally, the root mean square roughness σ and the correlation length τ are used to characterize the morphologies of the rough surfaces. The root mean square roughness represents the standard deviation of the height distribution and the correlation length denotes the distance above which the heights of two points are statistically uncorrelated, respectively [19]. While utilizing EMA to model the rough surface in ellipsometry, the surface roughness layer is replaced by a single homogeneous and flat layer. Therefore, compared to actual surfaces, the results calculated by the EMA model are not always high-precision.

**Figure 4:**
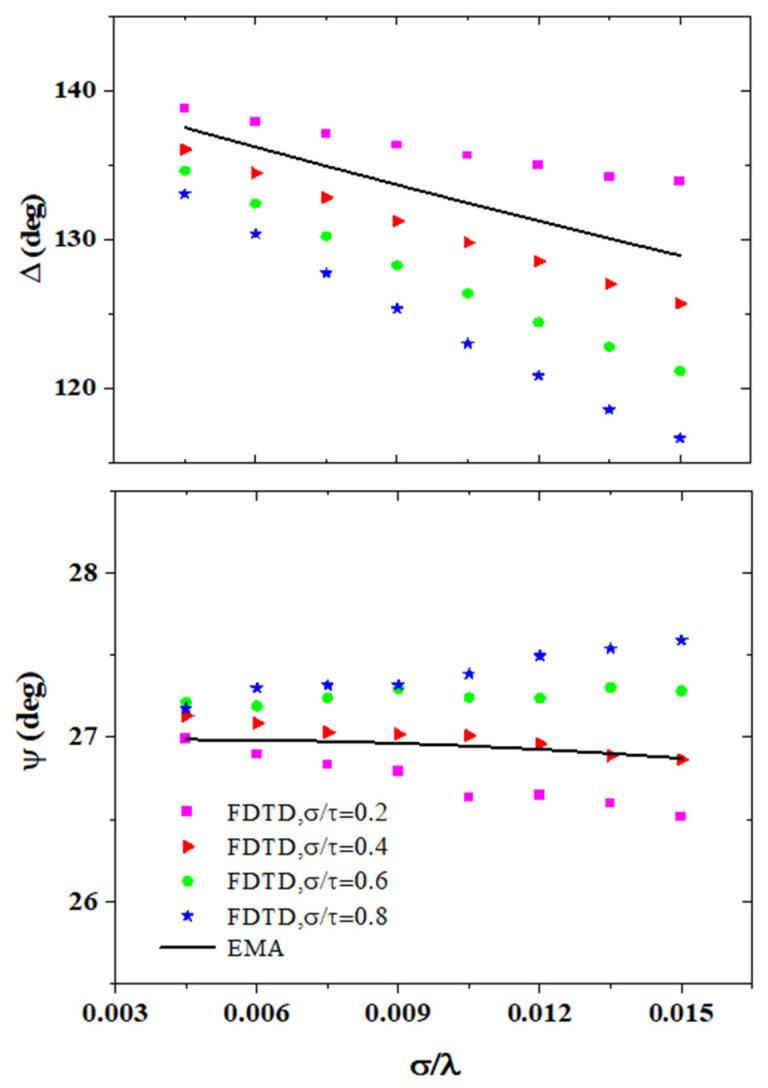
Comparison of the SE parameters between FDTD and EMA.

In order to evaluate the precision of the EMA model intuitively, we chose the factors affecting the precision of the EMA model as coordinates and drew the regime maps. The comparisons of the SE parameters between the actual surfaces and the EMA model are shown in Figure 4. The results of the actual surfaces are simulated by the FDTD method. While keeping the root mean square roughness σ of 0.006 μm unchanged, we changed the correlation length τ and incident wavelength λ to investigate the variations of the SE parameters Ψ and Δ with σ/λ and σ/τ. We found that the precision of the EMA model changed with σ/τ and σ/λ and the error increased as σ/λ was increased. Therefore, the precision of the EMA model was related to the incident wavelength and morphologies of the rough surfaces and σ/λ and σ/τ were chosen to be the horizontal coordinate and vertical coordinate of the regime map of the EMA model, respectively.

### 3.3. Regime Map of the SE Parameters Errors Calculated by the EMA Model

To draw the regime map of the SE parameters errors calculated by the EMA model based on quantitatively calculated data, we defined the relative error of the SE parameters as:

(4) $$\delta \psi =\left|\frac{\psi_{EMA}-\psi_{FDTD}}{\psi_{FDTD}}\right|\times 100\%$$

(5) $$\delta \Delta =\left|\frac{\Delta_{EMA}-\Delta_{FDTD}}{\Delta_{FDTD}}\right|\times 100\%$$

where δΨ and δΔ are the relative errors of the amplitude ratio and the phase difference, respectively; $\psi_{EMA}$ and $\Delta_{EMA}$ denote the amplitude ratio and the phase difference of the EMA model obtained by the RCWA method; and $\psi_{FDTD}$ and $\Delta_{FDTD}$ represent the amplitude ratio and the phase difference of the actual surface calculated by the FDTD method. 

**Figure 5:**
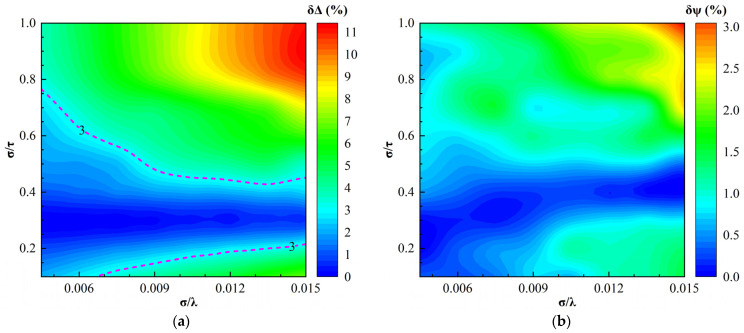
Regime map of SE parameters relative errors of the EMA model in the case of n = 3.2, k = 2.6, and σ = 0.006 μm: (**a**) regime map for the relative errors of the phase difference; (**b**) regime map for the relative errors of the amplitude ratio.

Figure 5 illustrates the regime maps of the SE parameter relative error δΨ and δΔ produced by using EMA to model the micro-rough surfaces whose optical constants n and k were 3.2 and 2.6, respectively, at any incident wavelength. As shown in Figure 5a, the maximum relative error of the phase difference δΔ was 11%. δΔ increased as the relative roughness σ/λ was increased under the condition of σ/τ remaining unchanged. In the area marked by pink dashed lines, the relative errors of the phase difference were within the range of 0 ≤ δΔ ≤ 3%, where the EMA may adapt to model the surfaces.

As shown in Figure 5b, the relative errors δψ of the amplitude ratio of the EMA model were small in the whole regime map and the maximum relative error was 3%. Therefore, using EMA to model the micro-rough surfaces with high absorption, we can obtain the amplitude ratio ψ with high precision. It also can be seen that δψ nearly increased as σ/λ increased on the condition of σ/τ being fixed. 

### 3.4. Regime Map of the Optical Constants Errors Inverted by the EMA Model 

To draw the regime map of the optical constants errors which were inverted by the EMA model based on quantitatively calculated data, we defined the relative error of the optical constants as:

(6) $$\delta n=\left|\frac{n_{EMA}-n}{n}\right|\times 100\%$$

(7) $$\delta k=\left|\frac{k_{EMA}-k}{k}\right|\times 100\%$$

where δn and δk are the relative errors of the refractive index and the extinction coefficient, respectively; $n_{EMA}$ and $k_{EMA}$ denote the refractive index and the extinction coefficient inverted by using the EMA model; and n and k are the constant values of 3.2 and 2.6, respectively, which represent the optical constants of actual rough surfaces.

**Figure 6:**
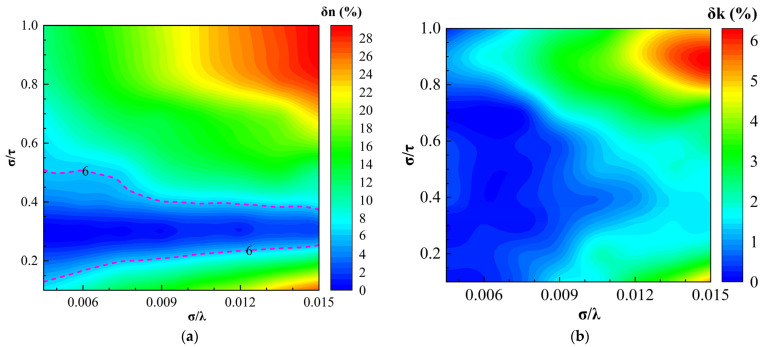
Regime map of the optical constants relative errors inverted by the EMA model in the case of n = 3.2, k = 2.6, and σ = 0.006 μm: (**a**) regime map for the relative error of the negative refractive index; and (**b**) regime map for the relative error of the extinction coefficient.

Figure 6 shows the regime maps of the optical constant relative error δn and δk produced by using the EMA model to invert the optical constants of the micro-rough surfaces of which n and k were 3.2 and 2.6, respectively, at any incident wavelength. As shown in Figure 6a, the maximum relative error of the refractive index δn was 29% and δn had the same variational rules as δΔ. In the area marked by the pink dashed lines, the relative errors of the refractive index were within the range of 0 ≤ δn ≤ 6%, where the EMA model may adapt to be used to invert the optical constants of the rough surfaces.

As shown in Figure 6b, the relative errors of the extinction coefficient δk were small in the whole regime map and the maximum relative error was 6%. That is to say, by using EMA to model the micro-rough surfaces with high absorption, we can invert the extinction coefficient k with high precision. It was also observed that δk decreased as σ/λ decreased on condition of σ/τ being constant. 

### 3.5. Effect of SE Parameter Error on Optical Constant Error 

The relative error distributions of the SE parameters and optical constants of the EMA model for the micro-rough surfaces whose optical constants n and k were 3.2 and 2.6, respectively, have been discussed by the regime maps in Section 3.3 and Section 3.4. Comparing the regime map in Figure 5a with that in Figure 6a, we observed that the distribution rules of the relative errors δΔ and δn were the same. Comparing the regime map in Figure 5b with that in Figure 6b, we realized that both of the relative errors δψ and δk were small in the entire investigated range. Therefore, we proposed that the relative error δn of the negative refractive index extracted by the EMA model was mainly caused by the phase difference relative error δΔ and the relative error δk of the extinction coefficient was mainly caused by the amplitude ratio relative error δψ.

**Figure 7:**
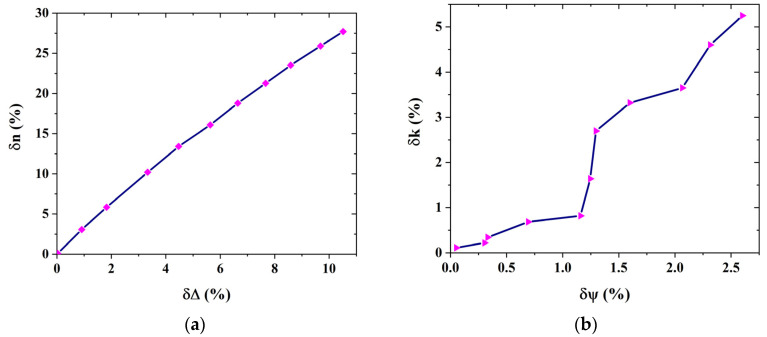
Relationship of the relative error between the ellipsometric parameters and optical constants of the EMA model: (**a**) relationship of the relative error between the phase difference and negative refractive index of the EMA model; and (**b**) relationship of the relative error between the amplitude ratio and extinction coefficient of the EMA model.

Next, we verified the above conjecture. We selected a few points randomly in Figure 5a and extracted the corresponding points in Figure 6a, which allowed us to obtain the relation between δn and δΔ, as shown in Figure 7a. It was observed that δn increased linearly as δΔ was increased. Similarly, we also randomly chose a few points in Figure 5b and extracted the corresponding points in Figure 6b, which enabled us to determine the relationship between δk and δψ, as shown in Figure 7b. We observed that δk increased as δψ was increased. Therefore, for the EMA model of the micro-rough surfaces with high absorption, the relative errors of the negative refractive index and extinction coefficient were mainly affected by the relative errors of phase difference and amplitude ratio, respectively, and the above conjecture was verified.

### 3.6. Effects of Root Mean Square Roughness on Regime Maps 

**Figure 8:**
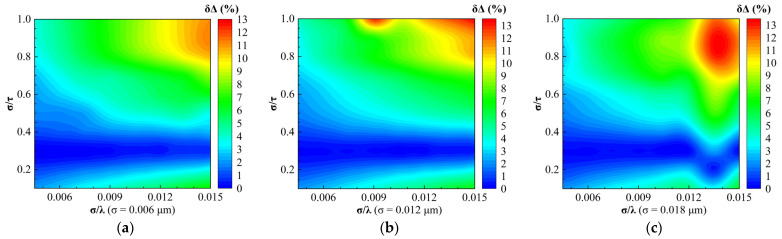
Regime maps for the relative error of the phase difference calculated by the EMA model for different root mean square roughness: (**a**) σ = 0.006 μm; (**b**) σ = 0.012 μm; and (**c**) σ = 0.018 μm.

**Figure 9:**
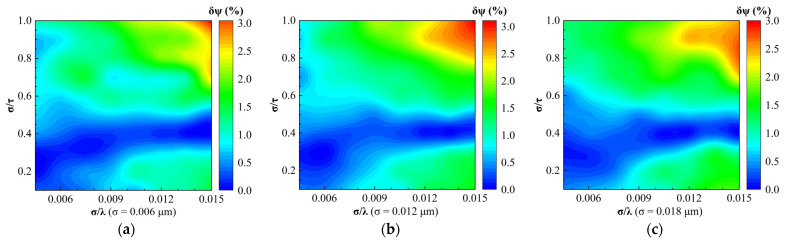
Regime maps for the relative error of the amplitude ratio calculated by the EMA model for different root mean square roughness: (**a**) σ = 0.006 μm; (**b**) σ = 0.012 μm; and (**c**) σ = 0.018 μm.

In order to investigate whether changing the root mean square roughness σ alone would have an effect on the regime maps of the relative errors of SE parameters δΔ and δψ calculated by the EMA model, respectively, we plotted the regime maps for different σ. As shown in Figure 8, the distributions of the phase difference relative errors δΔ for different σ were similar within the range of 0 ≤ δΔ ≤ 5%. Therefore, changing the root mean square roughness alone will not affect the regime maps of the phase difference error when the δΔ is no more than 5%. As shown in Figure 9, all the amplitude ratio relative errors δψ in the whole regime maps with different σ were no more than 3%. Although there were differences in some regions of the regime maps of δψ for different σ, we can assume that the regime maps of δψ do not change with the root mean square roughness changing alone. 

**Figure 10:**
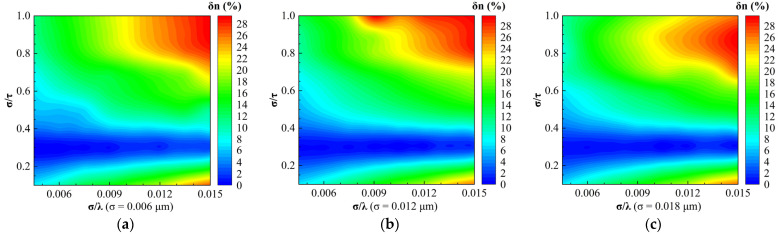
Regime maps for the relative error of the extinction coefficient inverted by the EMA model for different root mean square roughness: (**a**) σ = 0.006 μm; (**b**) σ = 0.012 μm; and (**c**) σ = 0.018 μm.

**Figure 11:**
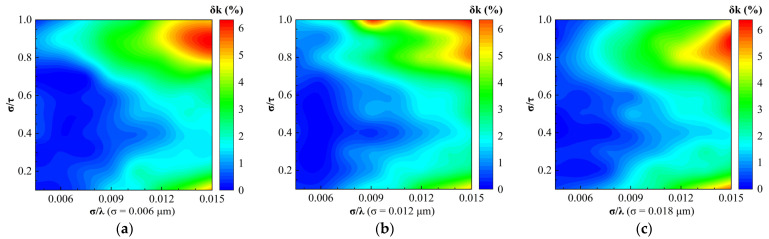
Regime maps for the relative error of negative refractive index inverted by the EMA model for different root mean square roughness: (**a**) σ = 0.006 μm; (**b**) σ = 0.012 μm; and (**c**) σ = 0.018 μm.

Figure 10 and Figure 11 show the regime maps of the relative errors of optical constants n and k inverted by the EMA model for different root mean square roughness, respectively. We observed that the distributions of the refractive index relative errors δn for different σ were similar within the range of 0 ≤ δn ≤ 16%. In addition, the regime maps of the extinction coefficient relative errors δk for different σ were similar, ignoring the differences in some regions. 

Therefore, we conclude that changing σ alone has little effect on the regime maps of the relative errors of SE parameters and optical constants.

## 4. Conclusions

In this paper, EMA is performed to model the micro-rough surfaces with the optical constants n and k of 3.2 and 2.6, respectively, and the film thickness and the volume fraction of the EMA model are σ and 50%, respectively. The SE parameters of the EMA model and micro-rough surfaces are simulated by the RCWA method and FDTD method, respectively. The FDTD method is validated with experimental results. Through observing the optical constants of W at the wavelength of 0.35 μm, we select the rough surface whose optical constants n and k are 3.2 and 2.6, respectively, which are kept unchanged with wavelengths to represent an actual rough surface with high absorption. By analyzing the factors that affect the accuracy of the EMA model, we choose σ/λ and σ/τ as the horizontal coordinate and vertical coordinate of the regime map, respectively. Then, the regime maps of the EMA model are drawn. The results show that using EMA to model micro-rough surfaces with high absorption can result in a higher precision of the amplitude ratio and extinction coefficient. The precisions of ψ, Δ, n, and k increase as the relative roughness σ/λ decreases. The precision of ψ has an influence on the precision of k and the precision of Δ affects the precision of n. Changing σ alone has little effect on the regime maps of the relative errors of SE parameters and optical constants. By drawing the regime map of the EMA model, one can clearly determine whether EMA adapts to model the rough surfaces or not. 

## Data Availability

Data is contained within the article.
